# DOSIF: Long-Term Daily SIF from OCO-3 with Global Contiguous Coverage

**DOI:** 10.3390/s25216771

**Published:** 2025-11-05

**Authors:** Longlong Yu, Xiang Zhang, Lizhi Wang, Rongzhuma Ga, Yingying Chen, Peng Cai

**Affiliations:** College of Computer Science, Chengdu University, Chengdu 610106, China; zhangxiang@stu.cdu.edu.cn (X.Z.); wlz3087964164@163.com (L.W.); grong99017601@163.com (R.G.); 19508146649@163.com (Y.C.); caipeng@stu.cdu.edu.cn (P.C.)

**Keywords:** SIF, daily, OCO-3, data driven, Google Earth Engine, global contiguous

## Abstract

Satellite-retrieved solar-induced chlorophyll fluorescence (SIF) provides an advanced proxy for global vegetation productivity. Recently, new high-quality remote sensing SIF datasets and reanalysis products have significantly advanced the application of SIF. However, the lack of long-term, daily resolution datasets continues to limit the precise exploration of vegetation dynamics, primarily due to challenges in daily modeling accuracy, substantial data volume, and computational demands. In this study, supported by the Google Earth Engine (GEE) platform, we developed a data-driven approach based on the Moving Spatial–Temporal Window Sampling (MSTWS) strategy for reconstructing long-term daily SIF. By learning the relationship between high-spatial-resolution Orbiting Carbon Observatory (OCO)-3 SIF and MODIS surface reflectance, we established a spatially and temporally specific daily prediction model for each day of the year (DOY), reconstructing the long-term daily OCO-3 SIF (DOSIF) from 2001 to the present with a global contiguous distribution. The prediction framework demonstrated robust performance with an R^2^ of 0.92 on the training set and 0.81 on the validation set, indicating strong predictive ability and resistance to overfitting. Systematic evaluation of the dataset showed that DOSIF accurately captures the expected spatiotemporal distribution patterns. Cross-sensor validation with independent airborne SIF measurements further enhanced the reliability of the DOSIF dataset.

## 1. Introduction

Solar-induced chlorophyll fluorescence (SIF) retrieved from satellite-based hyperspectral data provides a novel approach to monitoring global vegetation productivity [1,2,3]. Numerous studies have demonstrated that satellite-based SIF observations yield more accurate estimates of gross primary productivity (GPP) than traditional reflectance-based approaches [4,5,6,7,8,9,10,11]. This superiority of SIF lies in its direct linkage to the physiological process of photosynthesis, reflecting the actual intensity of photosynthetic activity [3,12]. In contrast, conventional indices such as the Normalized Difference Vegetation Index (NDVI), Enhanced Vegetation Index (EVI), and Near-Infrared Reflectance of Vegetation (NIRv) and structural metrics like the Leaf Area Index (LAI), which are widely used as proxies for vegetation productivity, are indirect indicators that reflect the potential for photosynthesis rather than its actual occurrence [5,7,13,14,15,16]. The successful retrieval of SIF via remote sensing has provided an unprecedented tool for monitoring vegetation productivity dynamics on large spatiotemporal scales. Currently, various satellites, including SCHIAMACHY, GOME-2, OCO-2, TanSat, OCO-3, TROPOMI, and the recently launched FLEX, are capable of providing SIF data [1,3,11,17,18,19]. However, due to the operational parameters and configurations of these satellites, the spatial and temporal resolution and completeness of the coverage vary significantly, leading to limitations in data quality. Early missions like SCHIAMACHY and GOME-2 were severely constrained by low spatial resolution and incomplete temporal coverage [1,20]. The advent of satellites such as OCO-2, OCO-3, and TanSat has substantially alleviated these limitations, particularly in spatial resolution [11,18,19]. The latest TROPOMI SIF data provide an unprecedented balance of resolution and coverage, offering products with daily temporal resolution and spatial resolution as fine as 1 km, significantly reducing constraints on remote sensing SIF applications [11,12,21,22,23,24,25].

Over the past 40 years, substantial remote sensing data closely related to vegetation indicators have accumulated, including canopy reflectance data, environmental indices, and various disaster and extreme climate event data. The potential value embedded in these historical data remains underexplored. These datasets may hold key insights into significant issues such as the mechanisms of climate change and vegetation productivity response, the impact of human activities on vegetation, and global vegetation productivity dynamics. In the era of big data and artificial intelligence, high-quality SIF datasets that coincide with these historical observations could further advance research in these areas [3,11,12,21,22,23,24,25]. However, the current satellite SIF data do not fully meet these needs due to their limited spatiotemporal coverage. For instance, OCO-2 and OCO-3 provide high-spatial-resolution data but are constrained by a 16-day revisit cycle and sparse spatial coverage [11,18,20,26,27]. Although TROPOMI represents advancements in SIF retrieval with improved spatial and temporal resolutions, challenges remain—particularly regarding missing observations and inconsistencies in data quality. Moreover, the relatively short time span of TROPOMI SIF data further limits their application for long-term historical analyses [17,27,28].

One viable solution to address these limitations is to reconstruct unobserved SIF signals across time and space using data-driven models based on available satellite SIF data. Over the past few years, numerous SIF reconstruction datasets have been developed, significantly mitigating some of the data-quality constraints. For example, the SIF_OCO2_005 product provides globally continuous coverage, addressing the issue of spatial gaps in OCO-2 data [26]. However, its 16-day temporal resolution and the inherent temporal limitations of satellite data remain unaddressed. Long-term datasets such as SIF_005 and GOSIF, which span over 20 years, offer valuable records but are limited to monthly or 8-day temporal resolutions, which are insufficient for short-term vegetation stress studies [20,29]. In such cases, tasks with high temporal resolution requirements often resort to temporal interpolation or curve-fitting methods, which can compromise data certainty. Therefore, a dataset that simultaneously meets the demands for extended temporal coverage and high temporal resolution would significantly advance the application of SIF data in vegetation productivity dynamics research.

However, establishing a daily SIF prediction model presents several challenges: (1) The vast amount of feature data, including multiband surface reflectance and various reanalysis datasets commonly used in data-driven models, leads to an enormous data volume in long-term daily SIF reconstruction tasks. (2) There are significant computational demands, as the large data volume requires substantial computational resources for effective model training and prediction, potentially exceeding those of conventional data-driven tasks. (3) There is a trade-off between model specificity and robustness, which is perhaps the most critical challenge [20,26]. Given the primary goal of daily resolution data reconstruction—to maintain the authenticity of data under specific spatiotemporal conditions—the challenge is to balance model robustness with specificity [26]. Broad models in machine learning may offer stable performance but often lose spatiotemporal specificity, while models trained on highly focused data may suffer from overfitting and lack diversity due to the limited scope of the training data, compromising robustness. This is a key consideration for studies attempting spatiotemporal-specific modeling [20,26].

In light of these challenges, we aim to develop a data-driven framework that balances model specificity and robustness for reconstructing a globally consistent, long-term, and high-spatiotemporal-resolution SIF product at a daily scale. Specifically, we pursue the following objectives: (1) to generate a daily OCO-3 SIF (DOSIF) dataset spanning from 2001 to the present at a spatial resolution of 0.05°; (2) to systematically evaluate the reconstruction framework, including a comparative analysis of machine learning algorithms to empirically determine and justify the final choice of algorithm, an assessment of performance across diverse biomes to demonstrate stability, and benchmarking of the proposed Moving Spatial–Temporal Window Sampling (MSTWS) strategy against a universal modeling approach to validate its effectiveness; (3) to rigorously validate the prediction accuracy of DOSIF through standard machine learning validation, visual assessment of spatial patterns, and cross-sensor evaluation with independent airborne SIF measurements.

## 2. Materials and Methods

### 2.1. Data

Our overall approach is to apply a data-driven method to establish a model capable of predicting historical SIF values on a daily basis and systematically evaluate its performance. To achieve this goal, we require access to target SIF values, feature data for the model, spatiotemporal control data, and data for cross-sensor validation. Table 1 summarizes all datasets used in this study along with their key attributes. The following subsections describe the details of each dataset.

#### 2.1.1. SIF Data

In this study, we used the latest OCO-3 SIF data from 2019 to 2023 as the target variable. The OCO-3 satellite provides high-quality solar-induced chlorophyll fluorescence (SIF) products at a spatial resolution comparable to our target of 0.05 degrees. Its sample size and spatiotemporal coverage adequately meet the requirements for our target data. Furthermore, OCO-2/OCO-3 SIF data have been widely adopted as benchmark sources for several existing reconstructed SIF datasets—such as SIF_005, GOSIF, and CSIF—whose applicability in data-driven modeling is well established [20,26,29,30].

The original OCO-3 SIF dataset includes variables such as ‘SIF_740nm’, ‘Daily_SIF_740nm’, ‘Daily_SIF_757nm’, and ‘Daily_SIF_771nm’. Here, ‘SIF_740nm’ denotes the instantaneous SIF intensity at 740 nm, while the ‘Daily_SIF_*’ variables represent the corrected daily mean SIF values, which exhibit significantly lower uncertainty compared to the instantaneous retrievals and are thus more suitable for modeling. To ensure high data quality, we used the daily SIF variables as they align better with our modeling objectives.

We implemented a three-step procedure to further screen high-quality observations and reduce uncertainties:

1. We first applied the quality control flag provided in the original OCO-3 SIF dataset to filter out lower-quality retrievals, retaining only the highest-quality data.
2. We then performed cross-band noise reduction following the method of Sun et al. [12], which effectively combines SIF observations at 757 nm and 771 nm to suppress random noise. The formula is given as

(1) $$SIF_{daily}=0.5\times (SIF_{757nm}+1.5\times SIF_{771nm})$$

3. To effectively reduce noise in SIF at the footprint scale that could impact the model, we further applied a five-nearest-neighbor smoothing technique, as suggested by Yu et al., to generate training data [26]. The following formula illustrates this method.
  (2) $$SIF_{smoothed}=0.2\times \sum_{i=1}^{5}SIF_{daily_{i}}$$
  where $SIF_{daily_{i}}$ represents the *i*-th daily observation among the five nearest neighboring footprints to the target SIF footprint. According to Yu et al., the five-nearest-neighbor footprints align well with the target resolution, and the processed data can enhance the model’s future stability [26].

#### 2.1.2. MODIS Surface Reflectance

To accurately match the daily scale OCO-3 SIF data during model training and to provide a high-quality daily resolution feature set during prediction, we utilized MODIS Bidirectional Reflectance Distribution Function (BRDF)-corrected 7-band surface reflectance. MCD43A4 offers daily 500 m resolution data with over 20 years of temporal coverage, making it the only data source capable of providing effective features without the need for reanalysis [30]. During the modeling phase, all 500 m resolution MCD43A4 pixels within the SIF footprint will be averaged to match the target variable for training. During the prediction phase, MCD43A4 will be resampled to 0.05 degrees to produce the reconstructed SIF dataset. All seven bands of data will be used for both training and prediction.

#### 2.1.3. Land-Cover Data

We used the annual MCD12C1 Version 6 land-cover data, which are globally available at a 0.05-degree (approximately 5.6 km) spatial resolution. This native resolution aligns with our target grid for the DOSIF product [31]. Specifically, we used the International Geosphere-Biosphere Programme (IGBP) classification system within the MCD12Q1 dataset along with location information to guide the spatial division of the model. The modeling and prediction processes were applied only to vegetated areas, while non-vegetated regions such as water bodies, snow and ice, deserts, and impervious surfaces were excluded.

#### 2.1.4. CFIS SIF Data for Cross-Sensor Evaluation

The Carbon Flux Imaging Spectrometer (CFIS) mission is an independent airborne SIF dataset, offering high-fidelity, direct observations of solar-induced chlorophyll fluorescence, making it an ideal benchmark for evaluating the effectiveness of our data-driven reconstruction framework under the MSTWS strategy [11]. This study used all CFIS campaigns conducted in 2016 to validate DOSIF, where it was co-located with CFIS flights. To achieve this, we spatially aggregated the CFIS SIF to match the 0.05° resolution of DOSIF. CFIS retrieves SIF at 755 nm, which is roughly 12% higher in magnitude than OCO-2’s 757 nm [32]. We thus applied a 1/1.12 scaling factor to CFIS SIF prior to comparison with DOSIF. The following formula illustrates this method.

(3) $$SIF_{CFIS\_corrected}=\frac{SIF_{CFIS\_daily}}{1.12}$$

### 2.2. Methods

#### 2.2.1. Modeling Strategy

Our data reconstruction framework is based on establishing a mapping relationship between SIF and surface reflectance. Two principal strategies can be employed for this purpose: the universal strategy and the spatiotemporal-specific strategy.

The universal strategy involves constructing a general model using either all available samples or a sufficiently large random subset that meets the model’s requirements and computational expectations. Once trained, such a model can predict SIF values at any spatiotemporal location. This approach is computationally efficient, as it requires only a single training procedure to support all subsequent predictions. It also benefits from a large and diverse sample pool, which enhances the model’s generalization ability and stability. However, a major limitation of this strategy is its neglect of spatiotemporal specificity in the SIF–reflectance relationship, which can vary with vegetation type and environmental stress. To preserve robustness, the universal model inevitably sacrifices a certain degree of this specificity.

In contrast, the spatiotemporal-specific strategy involves sampling within defined spatial and temporal domains to capture context-dependent mapping patterns, thereby enabling more accurate predictions under specific conditions—an approach whose effectiveness has been validated in earlier studies. In previous work, sampling was generally conducted at fixed intervals aligned with the target temporal resolution, such as monthly or 16-day composites. Since the target datasets in those studies had relatively coarse temporal resolutions, this method could still ensure adequate sample size and diversity and thus balance specificity with generalization.

While the spatiotemporal-specific strategy is recognized for its superior performance and is adopted in this work, its direct application is challenged by the daily resolution of the DOSIF target. At this fine temporal scale, the samples from OCO-3 are inherently limited in number and diversity and are insufficient for constructing multiple robust models across different spatiotemporal divisions. Therefore, we propose a Moving Spatial–Temporal Window Sampling (MSTWS) strategy, which focuses on predicting DOY-specific SIF values. Instead of attempting to model every single day from 2001 to the present, we model each DOY (day of the year). Each DOY model can then be used to predict the SIF for the same DOY in any year.

Specifically, by using a moving temporal window, we collect data from a total of 16 days around each target DOY (i.e., the target DOY itself, the 7 days before, and the 8 days after) across all available years. These samples serve as the training data for that specific DOY. We further refine this approach by applying the sub-biome-level spatial division strategy proposed by Yu et al. (as detailed in Table 2) to conduct spatiotemporal-specific training [28]. This method integrates data from multiple years, ensuring an adequate sample size while moderately increasing sample diversity. It achieves a balance between model specificity and robustness, thereby enhancing the stability of the model’s performance. The MSTWS strategy, with its combination of temporal and spatial controls, effectively provides high-quality training samples for the daily prediction model.

**Figure 1:**
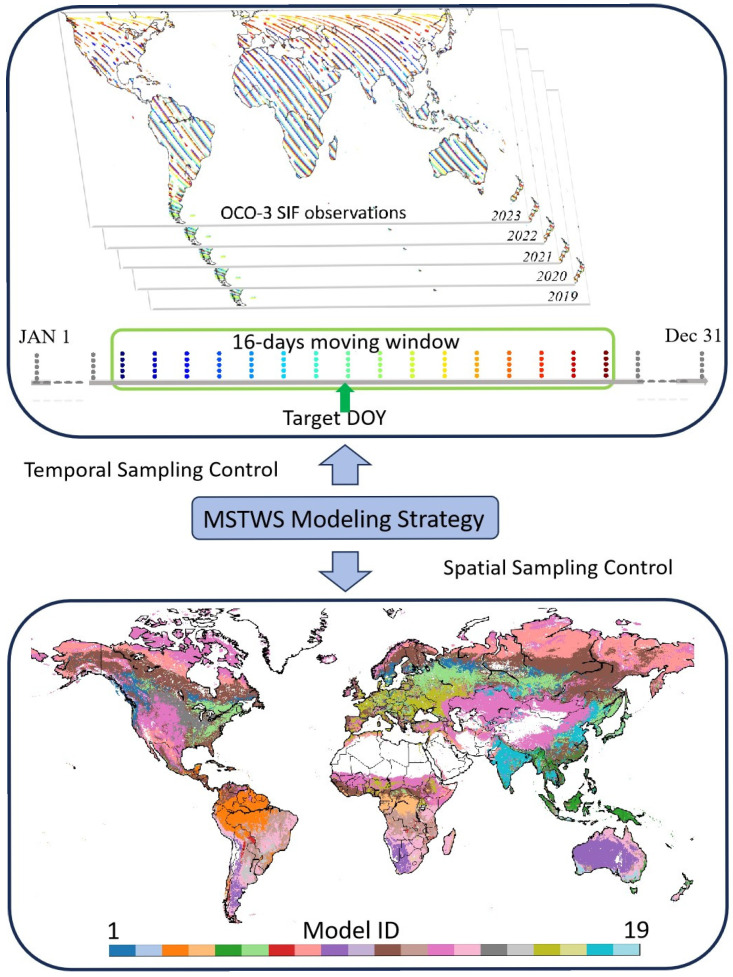
MSTWS strategy of sampling in the reconstruction of DOSIF.

For example, in the modeling process for DOY September 1st, the SIF data used include all available data between August 25th and September 9th from all years (2019 to 2023) within the temporal window. Figure 1 illustrates the implementation process of the MSTWS strategy.

#### 2.2.2. Machine Learning Algorithm Selection

In the reconstruction of SIF data, Artificial Neural Networks (ANNs) and Random Forest (RF) are the most commonly used methods. Many existing datasets have utilized one of these two approaches, such as SIF_005 [20], which employs RF, and SIF_OCO2_005, which utilizes an ANN [26]. The ANN is a powerful tool for modeling complex non-linear relationships, making it well-suited for tasks involving large, high-dimensional datasets like SIF [20,26,33]. It learns directly from data, automatically adjusting its internal parameters to minimize prediction errors. RF, on the other hand, is an ensemble learning method that combines multiple decision trees to improve prediction accuracy and robustness [34]. It is particularly effective in handling data with a large number of features and can provide estimates of feature importance [20].

CatBoost is a relatively new gradient-boosting algorithm that has gained attention for its ability to control overfitting and adapt well to diverse datasets. It is particularly effective in handling categorical features and can achieve high accuracy with less tuning compared to other boosting algorithms. CatBoost’s ability to provide robust predictions with fewer adjustments makes it increasingly popular in geoscientific variable prediction tasks [35,36].

In this study, Artificial Neural Networks (ANNs), Random Forest (RF), and CatBoost were implemented as competing candidate algorithms. Our objective was not to demonstrate the inherent superiority of any specific algorithm, but to establish a robust technical workflow that systematically evaluates multiple algorithmic options. Through an automated hyperparameter tuning process, the optimal algorithm–parameter combination was determined based exclusively on cross-validation performance. This approach ensures that selection is objectively guided by empirical validation results rather than predetermined assumptions, thereby enhancing the reliability of the modeling framework.

#### 2.2.3. Training and Prediction Configuration

For samples selected through the MSTWS strategy, a random subset of 5000 samples was chosen for training. If the available samples numbered fewer than 5000, all samples were used. In cases where the sample size was too small (fewer than 100), model training was not performed. If any models were not trained due to insufficient samples after training the 19 models for each DOY, a universal model was trained using the combined data for that DOY as a substitute for predictions. The dataset was split into training and validation sets at a 70:30 ratio.

During model training, a 5-fold cross-validation approach was employed to optimize model hyperparameters, ensuring that the most suitable configuration was found for future SIF predictions. For the ANN model, the hyperparameters tuned included the number of hidden layers, number of neurons per layer, learning rate, activation function, and regularization parameters. For the RF model, hyperparameters included the number of trees, maximum depth of each tree, minimum samples split, maximum features considered for splits, and bootstrap options. For the CatBoost model, hyperparameters included the number of boosting iterations, learning rate, depth, L2 regularization, and the use of one-hot encoding for categorical features. The best-performing model identified through cross-validation was used for the final SIF predictions for the specific DOY and corresponding spatiotemporal context.

To control the robustness of a specific model, we only train a model if its sample size is more than 1000. If the number is less than 1000, we ignore it. When predicting, the ignored spatiotemporal models will be replaced by the nearest-date model with the same spatial configuration.

#### 2.2.4. Evaluation Methods

To systematically and comprehensively evaluate the generated data, we performed machine learning regression assessments using reserved test samples that were not involved in the training process. The evaluation metrics included the root mean square error (RMSE) and the coefficient of determination (R^2^). Additionally, we conducted visual assessments of spatial distribution patterns and temporal series to further evaluate the data. Finally, we compared predicted SIF with independent airborne measurements from CFIS, which was specifically designed by the OCO-2 team to validate OCO-2 SIF retrieval.

#### 2.2.5. Cross-Biome Performance Assessment

To evaluate the performance of the proposed framework across different ecological contexts, we conducted a comprehensive assessment using the sub-biome classification system presented in Table 2. The reserved validation dataset was systematically stratified according to these biome categories, enabling us to examine the predictive accuracy of all three candidate algorithms—ANN, RF, and CatBoost—within each distinct vegetation type.

This analytical approach provides critical insights into two key aspects: First, it reveals how prediction accuracy varies across different biomes, highlighting potential challenges in specific ecological contexts. Second, it facilitates a direct comparison of algorithmic performance under consistent evaluation conditions. Most importantly, this stratified validation allows us to objectively verify whether the automatically selected optimal algorithm demonstrates consistent robustness across diverse ecosystems, thereby validating the rationality of our algorithm selection process and the general applicability of our modeling framework.

#### 2.2.6. Strategy Comparison 

To quantitatively evaluate the effectiveness of the proposed Moving Spatial–Temporal Window Sampling (MSTWS) strategy, we conducted a systematic comparison with a universal modeling approach. The universal model was trained on all available samples aggregated across all dates and locations, representing a conventional data-driven methodology that prioritizes generalization over spatiotemporal specificity. Both strategies were implemented and evaluated under identical conditions to ensure a fair comparison: (1) using the same underlying data sources (OCO-3 SIF and MODIS MCD43A4 reflectance); (2) applying the same data-quality control procedures; (3) utilizing the same algorithm with consistent hyperparameter optimization; (4) evaluating performance on the same independent validation dataset.

The comparative analysis focused on multiple performance metrics, including the coefficient of determination (R^2^), root mean square error (RMSE), and slope of the regression line. This comparison was specifically designed to assess whether the additional complexity of the MSTWS strategy yields significant improvements in prediction accuracy and robustness.

#### 2.2.7. Computational Environment

**Figure 2:**
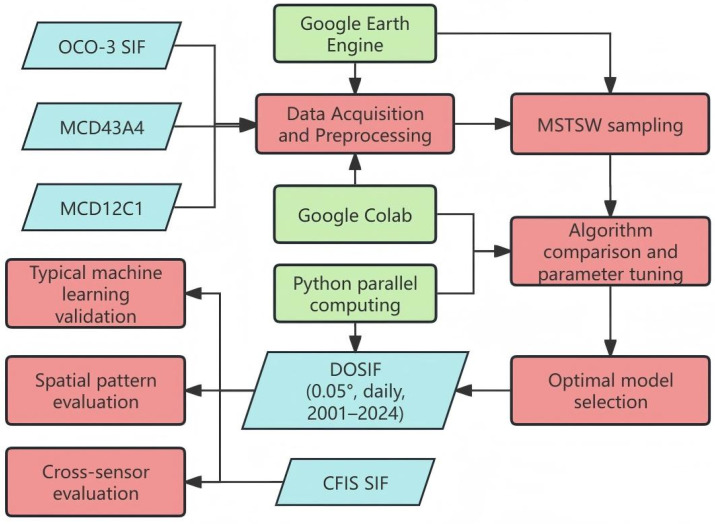
Workflow of the DOSIF reconstruction.

The task of processing daily data from 2001 to the present, particularly the seven bands of 500 m resolution MCD43A4 data, poses significant challenges for traditional data-processing methods. Fortunately, the Google Earth Engine (GEE) provides cloud-based data storage, object-based computation, and a highly concurrent computing environment [37,38,39]. All feature generation steps were completed using GEE, including feature matching for training data and the generation of global daily features spanning over 20 years. Data preprocessing, cleaning, and organization, as well as model training, were conducted in the Python 3.9 environment. Model training utilized parallel computing in Python and Google Colab [40]. The seamless integration of Python’s modeling capabilities with GEE’s cloud-based data handling and large-scale data processing ensured smooth operation of the entire framework. Figure 2 shows the workflow of the DOSIF reconstruction.

## 3. Results

### 3.1. Overall Performance of Prediction Framework

**Figure 3:**
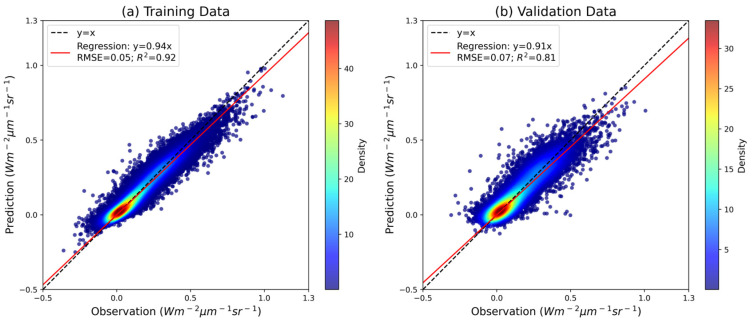
Performance of prediction framework: (**a**) training dataset; (**b**) validation dataset. The dataset was split into training and validation sets at a 70:30 ratio.

Through our predefined automated model selection and hyperparameter optimization framework, CatBoost was automatically identified as the final modeling algorithm for predicting DOSIF. The aggregated results from both the training and validation datasets are summarized in Figure 3. For the training set, the overall prediction statistics are R^2^ = 0.92, RMSE = 0.05 Wm^−2^ μm^−1^ sr^−1^, and slope = 0.94. For the validation set, the corresponding results are R^2^ = 0.81, RMSE = 0.07 Wm^−2^ μm^−1^ sr^−1^, and slope = 0.91. These results indicate that the model framework is able to achieve a consistently strong predictive performance under the MSTWS sampling strategy. Notably, the relatively small performance gap between the training and validation sets highlights CatBoost’s effectiveness in resisting overfitting and maintaining generalization across diverse conditions. This underscores the robustness and reliability of the proposed approach in reconstructing high-resolution, daily SIF data at the global scale.

### 3.2. Comparison of Machine Learning Algorithms Across Sub-Models

**Figure 4:**
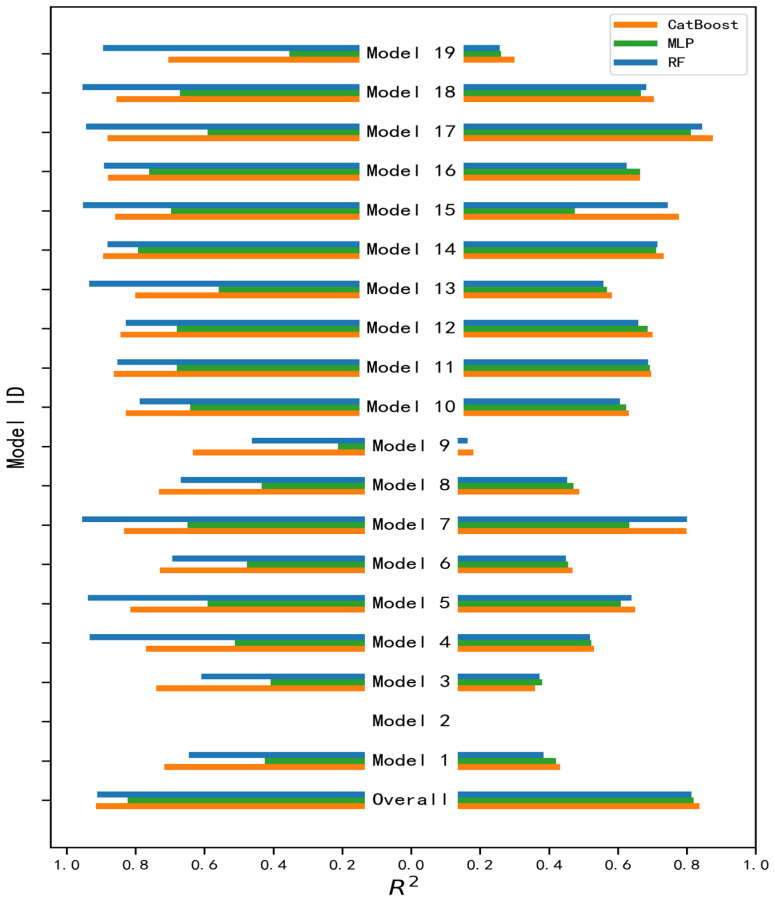
Comparison of machine learning algorithms across sub-models. For some models, data are missing due to insufficient training samples below our preset threshold.

Figure 4 presents the performance comparison of the three machine learning algorithms—Artificial Neural Networks (ANNs), Random Forest (RF), and CatBoost—across all sub-models defined in Table 1. The analysis reveals the following key findings.

The model’s performance varies across different sub-models in both training and validation datasets. While most sub-models exhibit good predictive capability, some vegetation types under specific spatiotemporal conditions show relatively poor performance. For example, croplands in Australia and shrublands in the southern hemisphere exhibit consistently lower R^2^ values across all three algorithms. This may indicate more complex or non-linear relationships between surface reflectance and SIF in these regions or phenological phases.

RF generally outperforms the other algorithms in terms of training accuracy, particularly for high-productivity vegetation types such as forests and croplands. For many sub-models, all algorithms achieve training R^2^ values above 0.90, but RF typically yields slightly higher scores, likely due to its ensemble learning structure and ability to capture complex patterns in the training data.

Despite slightly lower training performance in some cases, CatBoost demonstrates the best generalization ability across the majority of sub-models as evidenced by its superior validation R^2^ scores. This highlights CatBoost’s strength in resisting overfitting and maintaining robustness across diverse vegetation types and environmental conditions.

Certain models could not be trained due to data unavailability on specific days. This issue was anticipated in our methodology. To address this in the operational data production phase, we propose substituting missing models with those trained for the same sub-region and vegetation type from the nearest available day of the year (DOY).

Based on these comparative results and our framework’s emphasis on both predictive accuracy and model stability, CatBoost was selected automatically as the final machine learning algorithm for training each sub-model corresponding to each DOY in the production of the daily resolution SIF dataset.

### 3.3. Comparison Between the MSTWS and Universal Strategies

**Figure 5:**
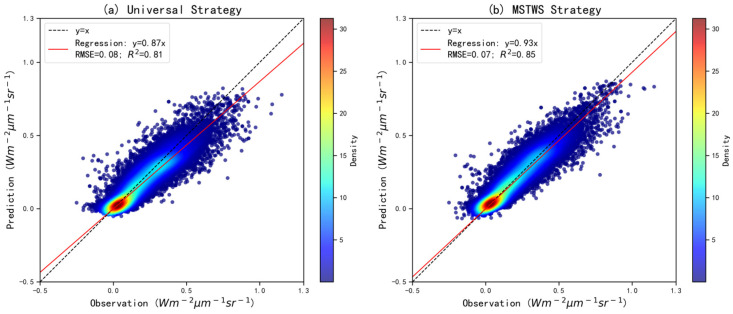
Performance comparison: (**a**) universal strategy; (**b**) MSTWS strategy.

To evaluate the effectiveness of the MSTWS strategy, we compared the predictive performance of two modeling approaches using the same reserved validation dataset: a universal model, trained on all aggregated samples regardless of date or location, and a DOY-specific model trained under the MSTWS strategy. The comparison results are shown in Figure 5. For the universal strategy, the overall prediction statistics are R^2^ = 0.81, RMSE = 0.08 Wm^−2^ μm^−1^ sr^−1^, and slope = 0.87, while for the MSTWS strategy, the results are R^2^ = 0.85, RMSE = 0.07 Wm^−2^ μm^−1^ sr^−1^, and slope = 0.93.

### 3.4. Spatial Pattern of DOSIF

**Figure 6:**
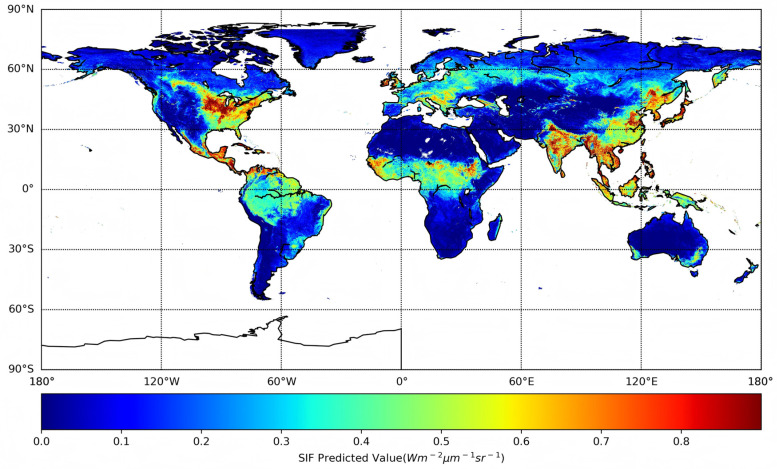
Spatial pattern of DOSIF on 1 August 2023.

As an illustrative example, Figure 6 shows the spatial pattern of DOSIF on 1 August 2023. This date corresponds to the peak of the growing season for most vegetation in the northern hemisphere. The high SIF values captured by DOSIF reflect the expected seasonal contrast between hemispheres and highlight regions of high vegetation productivity, including temperate and tropical croplands. Prominent examples include the U.S. Midwest Corn Belt and major agricultural zones in Asia, where photosynthetic activity often exceeds that of tropical forests during peak growth periods. Moderate SIF values are observed over deciduous broadleaf forests (DBFs) and high-latitude needleleaf forests (NFs), while low SIF values appear in arid and sparsely vegetated areas of the southern hemisphere during its austral winter. These spatial distributions are consistent with the patterns of gross primary productivity (GPP) reported in previous studies [20,26,41]. The spatial visualization of DOSIF on this date supports the prediction statistics discussed earlier and demonstrates the dataset’s ability to resolve fine-scale spatiotemporal variability at daily and 0.05-degree resolution.

### 3.5. Cross-Sensor Validation with Independent CFIS SIF

**Figure 7:**
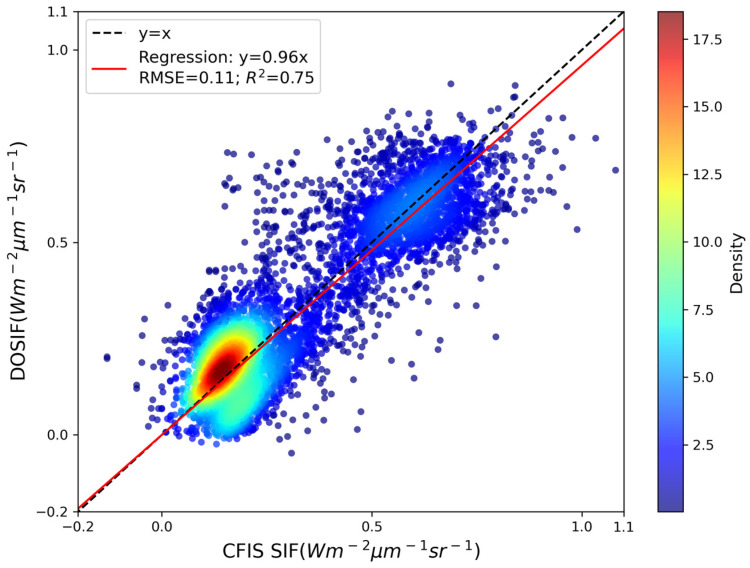
Cross-sensor validation using independent airborne CFIS SIF.

To further validate the accuracy and reliability of the reconstructed DOSIF dataset, we performed a cross-sensor comparison using independent airborne SIF measurements from the Carbon Flux Imaging Spectrometer (CFIS) mission. This airborne dataset offers high-fidelity, direct observations of solar-induced chlorophyll fluorescence, making it an ideal benchmark for evaluating the effectiveness of our data-driven reconstruction framework under the MSTWS strategy. The validation results are presented in Figure 7. The model achieved the following prediction statistics against CFIS observations: R^2^ = 0.75, RMSE = 0.11 W·m^−2^·μm^−1^·sr^−1^, and slope = 0.96. Given the challenging nature of cross-sensor validation—especially with high-precision airborne measurements—the obtained results are considered robust and satisfactory. These findings provide compelling evidence for the effectiveness of our MSTWS-based modeling approach in reconstructing long-term, daily resolution SIF with both spatial and temporal fidelity. The consistency with CFIS further reinforces the scientific credibility and practical utility of the DOSIF dataset.

## 4. Discussion

The successful implementation of this framework demonstrates the effectiveness of integrating cloud computing, parallel processing, and the novel Moving Spatial–Temporal Window Sampling (MSTWS) strategy for large-scale daily SIF reconstruction. Through seamless integration of the Google Earth Engine platform, Python-based parallel computing, and multi-task concurrency in Google Colab, this study establishes a technically feasible pathway to process massive spatiotemporal datasets and generate a globally contiguous daily SIF product (DOSIF) at 0.05° resolution spanning 2001–2024.

The overall prediction framework demonstrates robust performance, with R^2^ values of 0.92 (training) and 0.81 (validation), comparing favorably with existing SIF reconstruction datasets—particularly noteworthy given the more challenging task of daily resolution modeling. Cross-sensor validation with independent CFIS airborne measurements (R^2^ = 0.75) further confirms the framework’s effectiveness in capturing realistic SIF signals. The results clearly demonstrate the superiority of the MSTWS strategy over the universal modeling approach. The performance improvement can be attributed to the spatiotemporal specificity inherent in the MSTWS design, which enables context-aware modeling that better reflects local phenological patterns and vegetation–climate interactions through its DOY-specific and sub-biome-aware sampling strategy.

Among the three machine learning algorithms evaluated, CatBoost was automatically selected as the final model due to its consistently stable performance on the validation set, where it demonstrated stronger resistance to overfitting and better robustness across diverse conditions compared to ANN and RF, rather than any universal superiority. Cross-biome analysis reveals consistent variations in prediction accuracy across different vegetation types, with this pattern remaining stable across all algorithms. This phenomenon, also reported in previous studies, likely stems from the varying strengths of the SIF–reflectance relationship across different spatiotemporal contexts and biome characteristics [20,26]. Highly productive vegetation in temperate regions shows the best model performance—possibly because its reflectance exhibits a greater dynamic range across different SIF levels, making it more responsive to photosynthetic potential.

Uncertainties in the framework originate from two primary sources: inherent limitations in input data and methodological choices. All datasets employed—including OCO-3 SIF retrievals, MODIS surface reflectance, land-cover products, and CFIS observations—contain their own uncertainties that may propagate through the processing chain. While the five-nearest-neighbor smoothing of SIF data and spectral integration likely reduce random noise, the empirical scaling of CFIS data may introduce additional uncertainty in validation. Machine learning approximations also contribute inherent uncertainty. However, it should be noted that DOSIF is designed for regional-scale applications (0.05° grids) rather than point-level precision, making these uncertainties acceptable for its intended use in monitoring vegetation dynamics at landscape to global scales.

## 5. Conclusions

In this study, we developed a novel Moving Spatial–Temporal Window Sampling (MSTWS) framework to reconstruct a long-term, daily, and globally contiguous solar-induced chlorophyll fluorescence (SIF) product—DOSIF—at 0.05° spatial resolution from 2001 to present. The proposed framework effectively balances model specificity and robustness, enabling high-resolution SIF estimation with both spatial and temporal fidelity.

The following specific conclusions can be drawn from this work: (1) Machine Learning Algorithm Performance: Among the three evaluated algorithms—Artificial Neural Networks, Random Forest, and CatBoost—CatBoost demonstrated the most consistent and robust performance across diverse biomes and temporal conditions. Although it did not always achieve the highest training accuracy, its superior generalization capability and resistance to overfitting made it the most suitable algorithm for the global daily SIF reconstruction task. (2) Influence of Biome Type: Model performance varied considerably across different biome types. While most vegetation classes, particularly forests and croplands, showed a strong predictive accuracy (R^2^ > 0.85), certain regions with complex vegetation structures or under environmental stress—such as shrublands in the southern hemisphere and Australian croplands—exhibited relatively lower performance, highlighting the need for further refinement in these areas. (3) Comparison Between Modeling Strategies: The MSTWS strategy significantly outperformed the universal modeling approach, achieving an R^2^ of 0.92 compared to 0.85 for the universal model. This demonstrates the critical importance of incorporating spatiotemporal specificity in high-resolution SIF reconstruction, as the MSTWS strategy better captures local phenological patterns and vegetation–climate interactions. (4) Validation and Consistency: Comprehensive validation confirmed the reliability of the DOSIF dataset. The framework achieved an R^2^ of 0.88 on the independent validation set, and cross-sensor evaluation with airborne CFIS measurements yielded a strong agreement (R^2^ = 0.75). These results consistently support the capability of DOSIF to accurately capture spatiotemporal patterns of vegetation photosynthesis across scales.

The DOSIF dataset generated in this study provides a valuable resource for monitoring vegetation dynamics at high temporal resolution, particularly beneficial for detecting rapid vegetation responses to environmental stresses, tracking fine-scale phenological changes, and improving our understanding of carbon cycle dynamics. Future work will focus on incorporating additional environmental variables and refining the modeling approach for challenging biome types.

## Figures and Tables

**Table 1:** Datasets used in this study.

Data	Source	Spatial Resolution	Temporal Resolution	Spatial Coverage	Temporal Span	Role
Spaceborne SIF	OCO-3 SIF	1.3 × 2.25 km^2^	Daily	Striped local coverage	2019 to 2023	Target variable
Surface reflectance	MCD43A4	500 m	Daily	Global contiguous	2000–2024	Predictive features
Land cover	MCD12C1 Version 6	5600 m	Yearly	Global contiguous	2000–2024	Training and prediction configuration
Independent airborne SIF	CFIS SIF	<<0.05-degree	Daily	Regional	2016	Cross-sensor evaluation

**Table 2:** The sub-biome-level spatial division strategy.

Biome Types	Sub-Biomes	Model ID
NF	NH	1
	SH	2
EBF	Amazon	3
	Congo	4
	Southeast Asia	5
DBF	NH	6
	SH	7
SHR	NH	8
	SH	9
SAV	NH	10
	SH	11
GRA	NH	12
	SH	13
CRO	North America	14
	South America	15
	Europe	16
	Africa	17
	Asia	18
	Australia	19

NF: Needleleaf Forest. EBF: Evergreen Broadleaf Forest. DBF: Deciduous Broadleaf Forest. SHR: Shrubland. SAV: Savannas. GRA: Grassland. CRO: Croplands. NH and SH denote the northern and southern hemispheres, respectively. The Model ID is consistent throughout the text and all figures in this paper.

## Data Availability

Data sources: MODIS MCD43A4 https://www.earthdata.nasa.gov/data/catalog/lpcloud-mcd43a4-006 (accessed on 1 August 2025), OCO-3 SIF https://oco2.gesdisc.eosdis.nasa.gov/data/OCO3_DATA/ (accessed on 5 August 2025), MODIS land cover https://www.earthdata.nasa.gov/data/catalog/lpcloud-mcd12q1-061 (accessed on 1 August 2025), CFIS SIF ftp://fluo.gps.caltech.edu/data/CFIS (accessed on 3 July 2025). The DOSIF dataset is available from the corresponding author upon reasonable request.

## Abbreviations

The following abbreviations are used in this manuscript:

SIF	Solar-Induced Chlorophyll Fluorescence
MSTWS	Moving Spatial–Temporal Window Sampling
GEE	Google Earth Engine
CFIS	Carbon Flux Imaging Spectrometer
NDVI	Normalized Difference Vegetation Index
EVI	Enhanced Vegetation Index
NIRv	Near-Infrared Reflectance of Vegetation
LAI	Leaf Area Index
OCO	Orbiting Carbon Observatory
DOSIF	Daily OCO-3 SIF
DOY	Day of the Year
ANN	Artificial Neural Networks
RF	Random Forest
IGBP	International Geosphere-Biosphere Programme
NF	Needleleaf Forest
EBF	Evergreen Broadleaf Forest
DBF	Deciduous Broadleaf Forest
SHR	Shrubland
GRA	Savannas
CRO	Grassland
SH	Southern Hemisphere
NH	Northern Hemisphere
BRDF	Bidirectional Reflectance Distribution Function
